# RETRACTED: Kim et al. Neuroprotective Effect of Statins in a Rat Model of Chronic Ocular Hypertension. *Int. J. Mol. Sci.* 2021, *22*, 12500

**DOI:** 10.3390/ijms27156545

**Published:** 2026-07-23

**Authors:** Mi-Lyang Kim, Kyung Rim Sung, Junki Kwon, Go Woon Choi, Jin-A Shin

**Affiliations:** 1 Biomedical Research Center, College of Medicine, University of Ulsan, Asan Medical Center, Seoul 05505, Republic of Korea; iota76@gmail.com (M.-L.K.); goni8586@gmail.com (G.W.C.);; 2 Department of Ophthalmology, College of Medicine, University of Ulsan, Asan Medical Center, Seoul 05505, Republic of Korea; ssazzang1982@hanmail.net

The Journal retracts the article “Neuroprotective Effect of Statins in a Rat Model of Chronic Ocular Hypertension” [1].

Following the publication of the article and subsequent Correction [2], additional concerns regarding the figures were raised. The Editorial Office and Editorial Board therefore conducted a further assessment of the case, including a review of the materials previously provided by the authors.

During this assessment, the Editorial Office and Editorial Board determined that the available source materials were insufficient to fully verify the integrity of the figures and the associated Correction. Despite multiple requests, the authors did not provide the additional information and original data needed to address these concerns.

As the concerns could not be satisfactorily resolved, the Editorial Board has lost confidence in the reliability of the article’s findings and has decided to retract this article in accordance with MDPI’s retraction policy.

This retraction was approved by the Editor-in-Chief of the *International Journal of Molecular Sciences*.

The authors have been informed of this retraction but did not provide a comment on this decision.
